# Outcomes of radiation therapy for resectable M0 gastric cancer

**DOI:** 10.18632/oncotarget.22574

**Published:** 2017-11-03

**Authors:** Weipeng Gong, Hongwei Zhao, Shanshan Liu, Jie Guan, Xin Liu, Qingsheng Hou, Zhenyu Zhu, Hongliang Guo

**Affiliations:** ^1^ Department of Surgical Oncology, Shandong Cancer Hospital Affiliated to Shandong University, Jinan, Shandong Province, 250117, China; ^2^ Department of Radiation Oncology, Shandong Cancer Hospital Affiliated to Shandong University, Jinan, Shandong Province, 250117, China

**Keywords:** surgery, gastric cancer, radiation therapy, resectable without distant metastases

## Abstract

**Background:**

The role of radiaotion therapy in resectable gastric cancer patients without distant metastases remains controversial. This retrospective analysis was performed to identify whether resectable gastric cancer patients without distant metastases might benefit from radiation.

**Results:**

The results of the Kaplan-Meier analysis and log-rank test showed that a total of 3309 patients had a MST of 29.0 months, a 1-year survival rate of 74.7%, and a 3-year survival rate of 45.5%. Among them, the MST of the "RPS" group and the "RAS" group were significantly longer compared with that of the "No Radiation" group (32.7vs 32.9 vs 25.3 months, *P* < 0.05). The 1-year survival rates were 83.7%, 83.5% and 65.6% for the "RPS", "RAS" and "No radiation" groups, respectively (*P* < 0.05) and the 3-year survival rates were 52.6%, 63.6% and 44.9%, respectively (*P* < 0.05). The multivariate Cox proportional hazard regression analysis showed that radiation was an independent prognostic factor.

**Materials and Methods:**

A total of 5744 patients from the SEER database who were initially diagnosed with histologically confirmed gastric cancer without distant metastases from 2010 to 2013 were included. Patients were divided into three groups as follows: patients who underwent radiation after surgery ("RAS" group), patients who underwent radiation prior to surgery ("RPS" group) and patients who did not undergo radiation ,only surgery performed ("No radiation'"group).

**Conclusions:**

This retrospective analysis demonstrated that "RPS" or "RAS"alone were independent prognostic factors for survival improvement in selected gastric cancer patients without distant metastases.

## INTRODUCTION

Gastric cancer (GC) is the fourth most common type of cancer globally and the main cause of cancer-related mortality worldwide, specifically in Asian countries [1, 2]. An estimated 26,370 people will be diagnosed of gastric cancer and 10,730 people will eventually die of gastric cancer in the United States, in 2016 [3]. More than 70% of cases occur in developing countries, Eastern Asia (mainly in China) occupies half of the world total cases [4]. The survival of early primary gastric cancer patients has improved because of early tumor detection, curative surgical resection and adjuvant therapy. However, gastric cancer is often diagnosed at an locally advanced stage [5]. Complete resected with D2 lymphadenectomy is widely regarded as the standard of care [6]. However, even after D2 gastrectomy and effective adjuvant chemotherapy, locoregional recurrence and distant metastasis are remarkable problems, and the survival time is usually unsatisfactory. Researchers have begun to seek and explore new and more effective treatment options for locally advanced gastric cancer patients without distant metastases. The Intergroup 0116 (INT 0116) study suggested that adjuvant chemoradiotherapy after curative surgery Improved survival outcome [7] was reconfirmed in several studies [6, 8, 9].

Radiation therapy is used more commonly in treatment of gastric cancer at present [10]. However, in Eastern Asia countries D2 gastrectomy is accepted as a standard surgical procedure, adjuvant RT is not commonly given to completely resected patients [11, 12]. However lacking sufficient evidence from large randomized trials, adjuvant RT after D2 dissection remained controversy. In this study, we retrospectively analyzed whether gastric cancer patients without distant metastases might benefit from postoperative radiation therapy.

## RESULTS

### Patient characteristics

A total of 3309 eligible patients were included: 623 (18.8%) patients underwent radiation prior to surgery (“RPS” group), 1053 (31.8%) patients underwent radiation after surgery (“RAS” group). A total of 2766 (48.2%) patients were over 65 years old and 2144 (64.8%) were male. Patient characteristics and demographics are summarized in Table 1.

**Table 1:** The characteristics of patients with gastric cancer without distant metastases

Characteristic	Overall no. (%) (*N* = 3309)	Radiation prior to surgery	Radiation after surgery	No radiation	*p*
Age at diagnosis (y)	3309	623 (18.8)	1053 (31.8)	1633 (49.4)	< 0.001
≤ 65	2766 (48.2)	421 (24.1)	638 (36.5)	691 (39.5)	
< 65	2978 (51.8)	202 (13.0)	415 (26.6)	942 (60.4)	
Sex, *N* (%)	3309				< 0.001
Male	2144 (64.8)	511 (23.8)	647 (30.2)	986 (40.6)	
Female	1165 (35.2)	112 (9.6)	406 (34.8)	647 (55.5)	
Race, *N* (%)	3289				< 0.001
White	2265 (68.9)	558 (24.6)	613 (21.7)	1094 (48.3)	
Black	386 (11.7)	18 (4.7)	169 (43.8)	199 (51.6)	
Other	638 (19.4)	46 (7.2)	265 (41.5)	327 (51.3)	
T-stage *N* (%)	3284				< 0.001
T1a	19 (0.6)	6 (31.6)	3 (15.8)	10 (52.6)	
T1b	57 (1.7)	7 (12.3)	21 (36.8)	29 (50.9)	
T2	239 (7.3)	61 (25.5)	77 (32.2)	101 (42.3)	
T3	1795 (54.7)	499 (27.8)	515 (28.7)	781 (43.5)	
T4a	887 (27)	22 (2.5)	342 (38.6)	523 (59.0)	
T4b	287 (8.7)	19 (6.6)	91 (31.7)	17 (61.7)	
Tumor grade *N* (%)	3160				< 0.001
Well differentiated; Grade I	74 (2.3)	26 (35.1)	18 (24.3)	30 (40.5)	
Moderately differentiated; Grade II	736 (23.3)	191 (26.0)	208 (28.3)	337 (45.8)	
Poorly differentiated; Grade III	2262 (63.8)	331 (14.6)	765 (33.8)	2262 (63.8)	
Undifferentiated; anaplastic; Grade IV	88 (2.8)	18 (20.5)	31 (35.2)	39 (44.3)	
Nodal status, *N* (%)	3309				< 0.001
N0	431 (13.0)	134 (31.1)	95 (22.0)	202 (46.9)	
N1	1028 (31.1)	322 (31.3)	249 (24.2)	457 (44.5)	
N2	825 (24.9)	127 (15.4)	290 (35.2)	408 (49.5)	
N3	1025 (31.0)	40 (3.9)	419 (40.9)	566 (55.2)	
AJCC tumor stage	3309				< 0.001
IIB	904 (27.3)	203 (22.5)	253 (28.0)	448 (49.6)	
IIIA	860 (26.0)	255 (29.7)	238 (27.7)	367 (42.7)	
IIIB	793 (24.0)	102 (12.9)	300 (37.8)	391 (49.3)	
IIIC	752 (22.7)	63 (8.4)	262 (34.8)	427 (56.8)	

T-stage, N-stage and AJCC tumor stage according to the 7th edition of AJCC TNM staging.

### Survival analyses

The results of the Kaplan-Meier analysis and log-rank test showed that a total of 3309 patients had a MST of 29.0 months, a 1-year survival rate of 74.7%, and a 3-year survival rate of 45.5%. Among them, the MST of the “RPS” group and the “RAS” group were significantly longer compared with that of the “No Radiation” group (32.7*vs* 32.9 *vs* 25.3 months, *P* < 0.05).The 1-year survival rates were 83.7%, 83.5% and 65.6% for the “RPS”, “RAS” and “No radiation” groups, respectively (*P* < 0.05) and the 3-year survival rates were 52.6%, 63.6% and 44.9%, respectively (*P* < 0.05).

### Outcomes of the different subgroups

We compared the survival benefit of patients according to the subgroups, which accounted for age, gender, race, grade, T-stage, N-stage and AJCC tumor stage, by Kaplan-Meier analysis and log-rank test. Among the different subgroups, the survival benefits of patients in the “RPS” and “RAS” groups were better than those seen in patients in the “No radiation” group (Table 2). Specifically, in the age, race, T2, T3, T4a, T4b, IIIA, IIIB, Grade, N1, N2, N3, AJCC tumor stage, the results showed that the survival improvement of patients in the “RPS” and “RAS” groups was significantly higher compared with that of patients in the “No Radiation” group. Moreover, patients with stage IIB cancer in the “RAS” group had an increased survival benefit compared with those in the “RPS” group (*p* < 0.05). However, among those with stage IIIA, IIIB, IIIC cancer, no statistically significant differences were found in the MST, the 1-year or the 3-year survival rate among the “RPS” and “RAS” groups (*p* > 0.05) (Figure 3).

**Figure 1:**
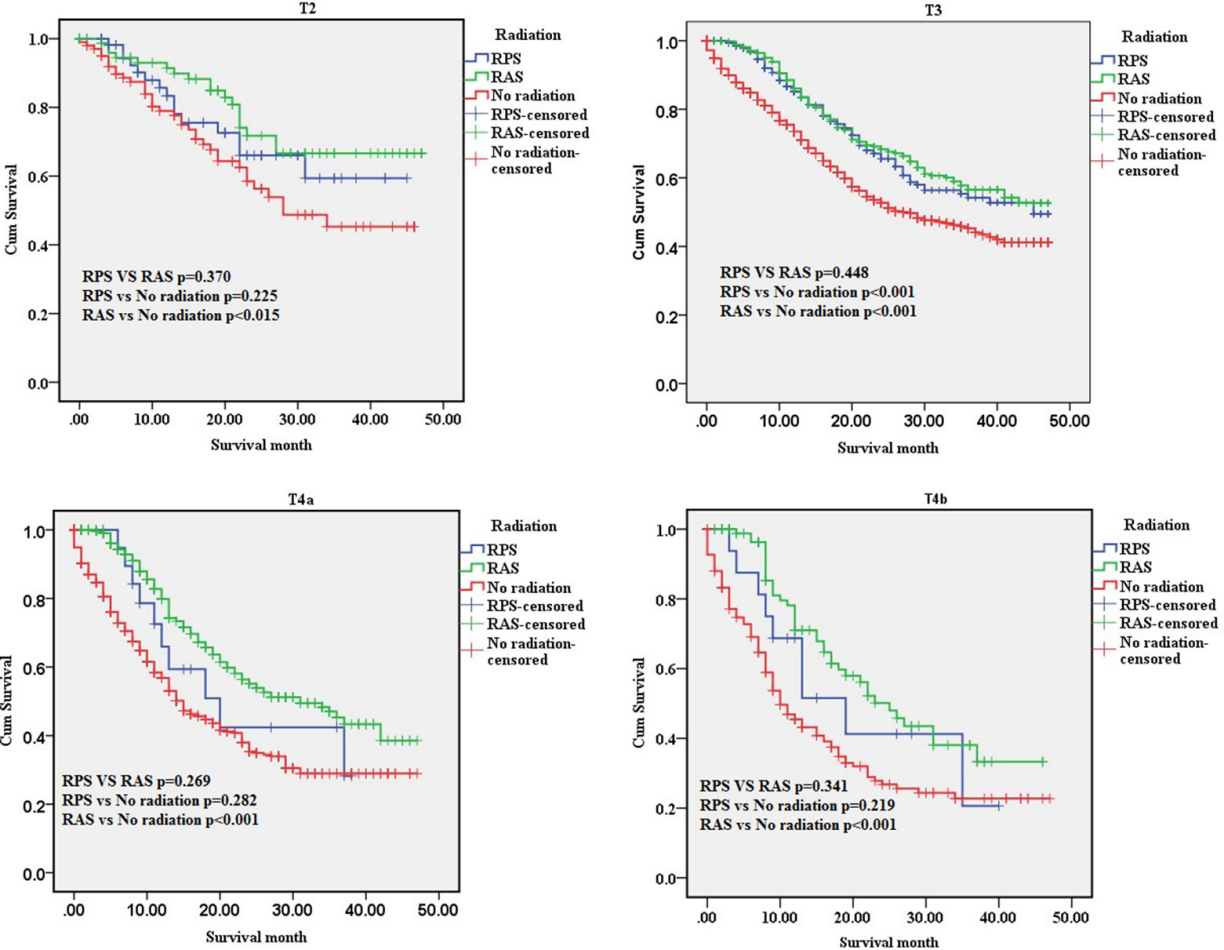
The survival curves of three groups in T-stage subgroups T2, T3, T4a and T4b subgroup. RPS: patients who underwent radiation after surgery; RAS: patients who underwent radiation prior to surgery; No radiation: patients did not undergo any radiation.

**Figure 2:**
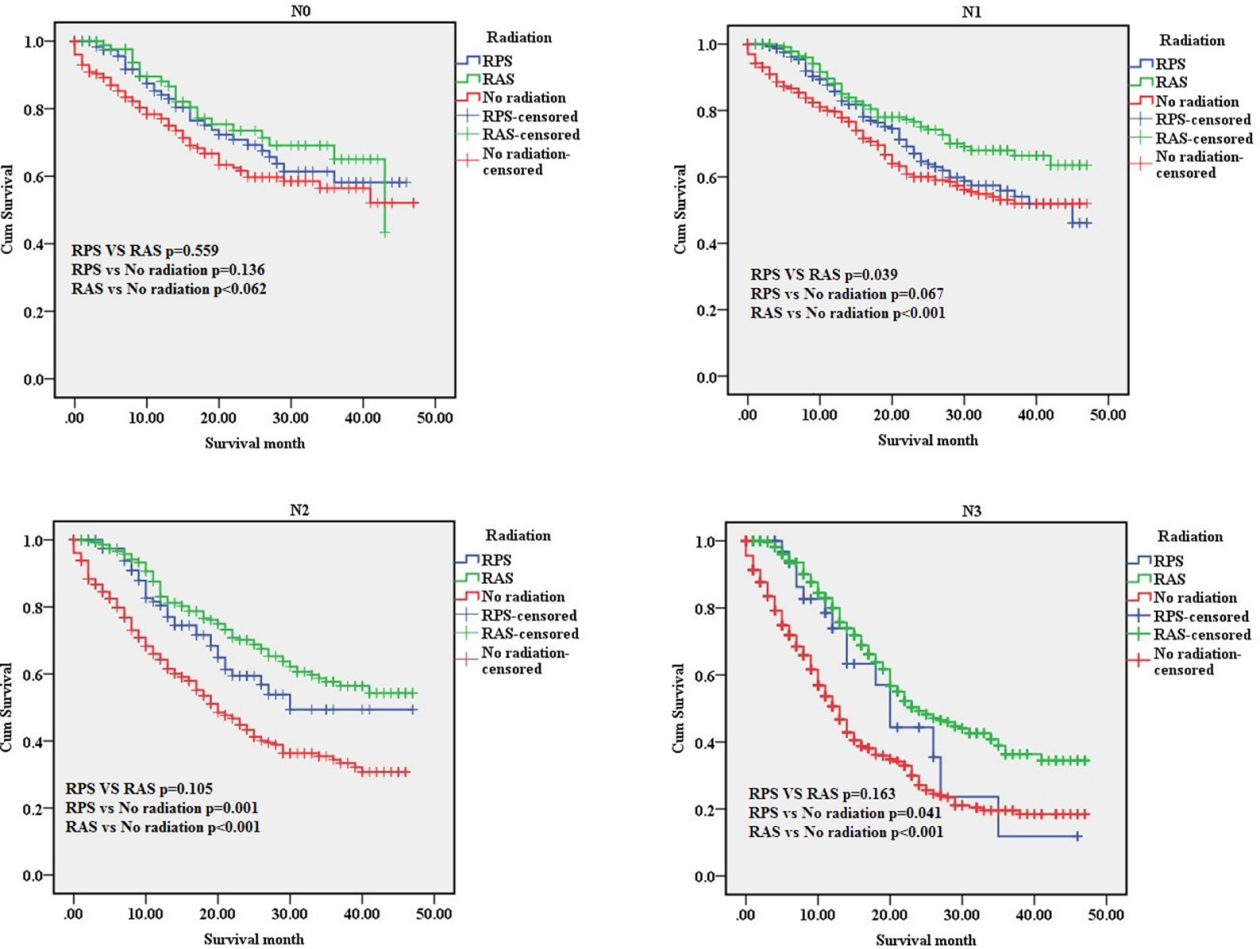
The survival curves of three groups in N-stage subgroups N0 subgroup; N1 subgroup; N2 subgroup; N3 subgroup. RPS: patients who underwent radiation after surgery; RAS: patients who underwent radiation prior to surgery; No radiation: patients did not undergo any radiation.

**Figure 3:**
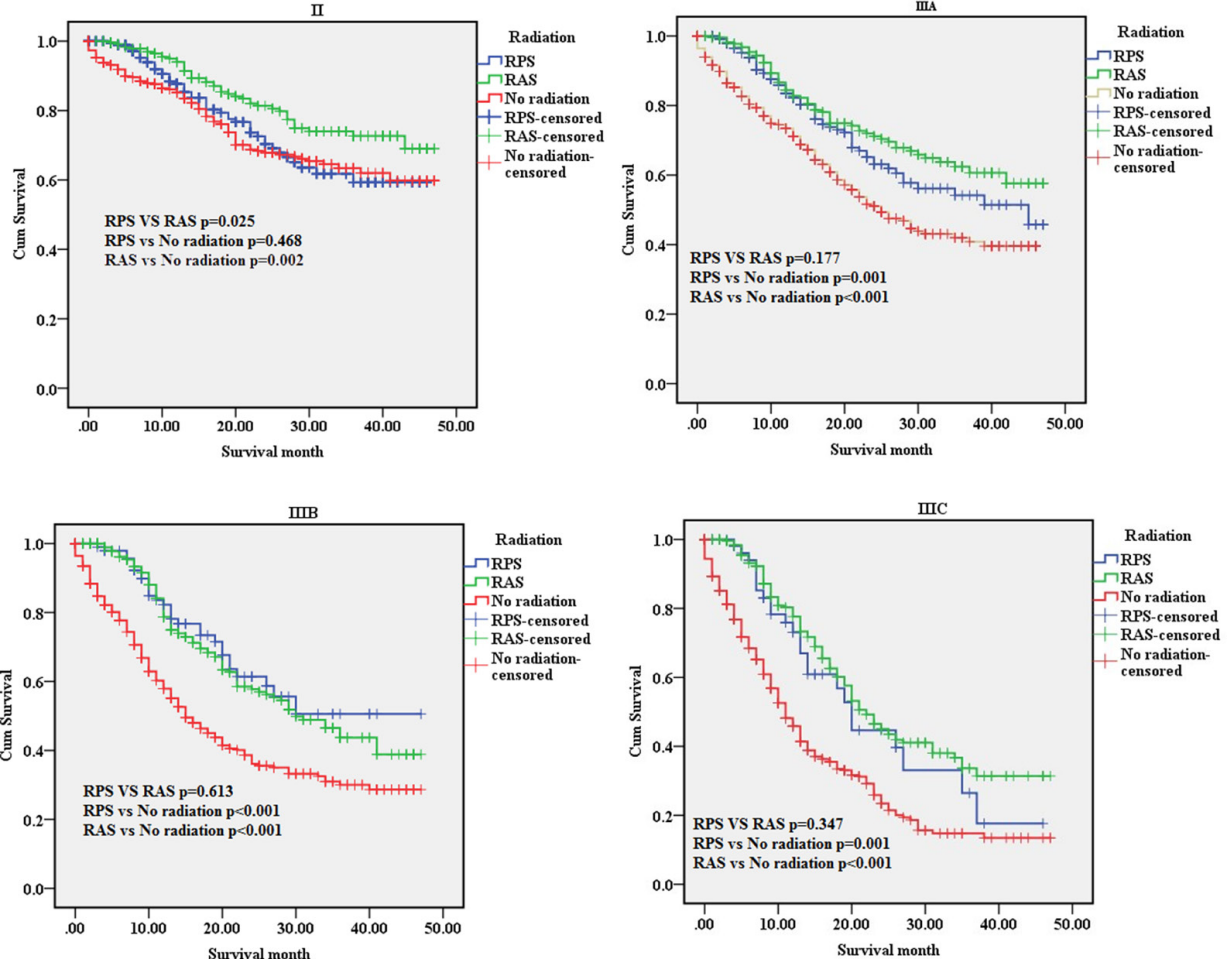
The survival curves of three groups in tumor stage subgroups II subgroup; IIIA subgroup; IIIB subgroup; IIIC subgroup. RPS: patients who underwent radiation after surgery; RAS: patients who underwent radiation prior to surgery; No radiation: patients did not undergo any radiation.

**Table 2:** Survival rate (%) and MST(Months) of patients with gastric cancer with distant metastases

	Radiation prior to surgery			Radiation after surgery			No radiation and/or cancer-directed surgery			
	Survival rate(%)		MST	Survival rate(%)		MST	Survival rate(%)		MST	
	1Y	3Y		1Y	3Y		1Y	3Y		*p*
**Total**	83.7	52.6	32.7	83.5	63.6	32.9	65.6	44.9	25.3	< 0.001
**Age at diagnosis (y)**										
≤ 65	88.6	54.8	33.6	84.0	54.9	33.5	75.2	46.5	29.5	< 0.001
< 65	77.3	47.3	30.6	82.7	50.0	31.8	58.5	31.8	22.2	< 0.001
**Sex,** ***N*** **(%)**										
Male	83.0	53.1	32.3	84.2	54.5	33.3	66.5	39.1	25.7	< 0.001
Female	86.8	52.0	32.7	82.5	50.7	32.2	64.1	36.3	24.6	< 0.001
**Race, *N* (%)**										
White	82.7	50.8	32.1	82.3	52.7	32.3	64.5	38.0	25.1	< 0.001
Black	90.9	72.7	33.2	78.8	41.1	30.0	60.4	35.8	23.6	< 0.001
Other	88.3	70.7	39.9	89.5	59.5	35.8	71.6	38.9	26.5	< 0.001
**T stage**										
T1a	NA	NA	NA	NA	NA	NA	NA	NA	NA	0.191
T1b	NA	NA	NA	NA	NA	NA	NA	NA	NA	0.259
T2	88.3	59.4	33.2	91.5	66.7	37.1	79.0	45.3	29.6	0.046
T3	85.1	54.2	33.4	86.0	56.6	34.3	73.5	45.2	28.3	< 0.001
T4a	66.0	28.3	23.1	79.8	45.3	30.0	56.8	28.9	21.5	< 0.001
T4b	68.8	20.6	21.6	71.0	38.1	27.0	45.5	22.8	18.2	0.001
**Tumor grade**										
Grade I–I	85.2	53.4	33.7	85.9	56.0	35.5	64.5	38.0	25.1	< 0.001
Grade III–IV	83.2	51.4	32.0	80.5	49.6	32.0	65.9	37.5	28.2	< 0.001
**N-stage,** ***N*** **(%)**										
N0	84.1	58.2	34.0	88.1	65.0	34.0	77.0	56.4	31.5	0.099
N1	85.8	55.9	33.5	88.1	68.0	36.9	79.6	53.1	31.4	0.001
N2	80.5	49.3	31.4	83.5	59.8	35.1	65.7	35.8	24.6	< 0.001
N3	73.9	11.8	22.4	80.0	36.4	28.2	50.6	19.6	18.2	< 0.001
**AJCC tumor stage**										
IIB	87.7	59.3	34.8	94.0	72.7	39.1	85.2	63.4	34.6	0.007
IIIA	83.5	54.2	32.9	84.5	62.4	35.3	73.4	42.0	27.0	< 0.001
IIIB	82.3	50.6	32.1	78.7	43.7	30.4	57.9	30.0	22.2	< 0.001
IIIC	73.1	26.5	23.7	77.6	33.6	26.7	45.8	14.8	16.3	< 0.001

The superiority in the “RAS” groups was significantly higher compared with the “No radiation” group in the IIB, T4a, T4b, N0, and N1 subgroups. However, no statistically significant differences were found among“RPS” group compared with the “No radiation” group.

### Multivariate analyses for survival

The multivariate Cox proportional hazard regression analysis showed that radiation was an independent prognostic factor (“RPS”, hazard ratio (HR) = 0.797, 95% confidence interval (CI) 0.650–0.977, *p <* 0.05; “RAS”, HR = 0.515, 95% CI 0.448–0.592, *p <* 0.001). We also analysed all the aforementioned factors in the subgroups and found that age, race, N-stage , T-stage and tumor stage were also independent prognostic factors (Table 3, Figures 1, 2).

**Table 3:** Multivariate analysis (Cox Proportional Hazard Model) of overall survival for patients with gastric cancer without distant metastases

	Wald	HR	95.0% CI for HR		*p*
			Lower	Upper	
Gender (male)	0.021	1.009	0.890	1.144	0.885
Race (white)	20.252				< 0.001
black	12.867	1.349	1.145	1.588	< 0.001
Other	18.645	1.608	1.296	1.995	< 0.001
Stage (IIIC)	9.744				0.021
IIB	4.309	0.640	0.420	0.975	0.038
IIIA	0.218	0.927	0.676	1.273	0.641
IIIB	0.131	0.961	0.776	1.191	0.717
T4b	27.569				< 0.001
T (T1a)	2.609	0.371	0.111	1.236	0.106
T1b	11.437	0.217	0.090	0.526	0.001
T2	11.628	0.477	0.312	0.730	0.001
T3	22.943	0.509	0.386	0.671	< 0.001
T4a	8.189	0.730	0.588	0.906	0.004
N (N3)	21.272				< 0.001
N0	11.056	0.509	0.342	0.758	0.001
N1	18.658	0.526	0.393	0.704	< 0.001
N2	17.035	0.654	0.534	0.800	< 0.001
Age (≤ 65)	51.663	0.644	0.571	0.726	< 0.001
Grade (I–II)	3.632	0.887	0.785	1.003	0.057
Radiation (No)	87.265				< 0.001
RPS	4.769	0.797	0.650	0.977	0.029
RAS	86.933	0.515	0.448	0.592	< 0.001

### Prognostic factors associated with radiation

**Figure 4:**
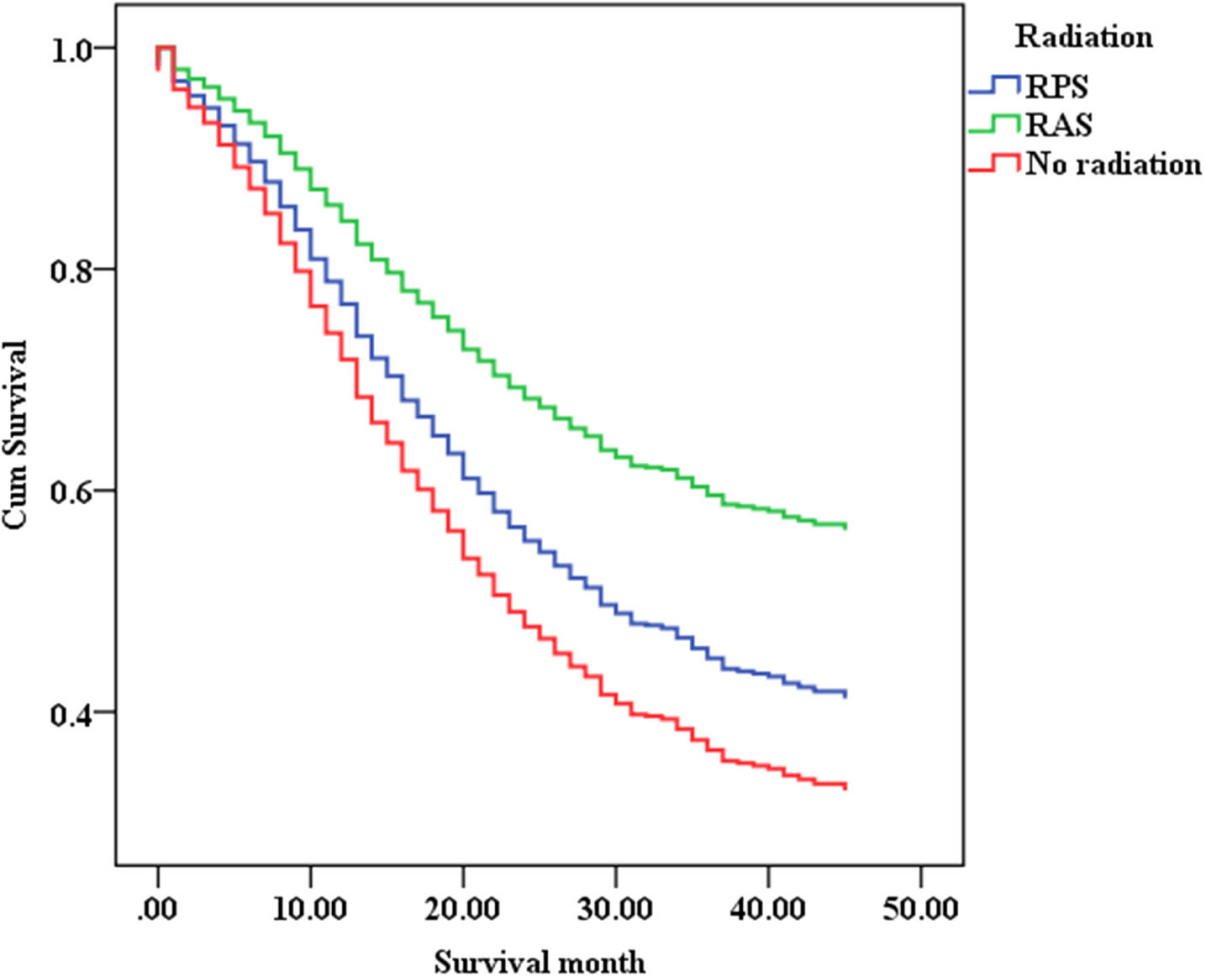
The multivariate survival curves of gastric cancer without distant metastases in different groups

The multivariate Cox proportional hazard regression analysis showed that in the “RPS” group, age was independent prognostic factors, while in the “RAS” group, race, tumor stage and grade were independent prognostic factors (Table 4 , Figure 4).

**Table 4:** Multivariate analysis (Cox Proportional Hazard Model) of overall survival of subgroups in group”RPS” group “RAS” and group “No radiation” respectively

	RPS					RAS					No rdiation				
	Wald	HR	95.0% CI for HR		*p*	Wald	HR	95.0% CI for HR		*p*	Wald	HR	95.0% CI for HR		*p*
			Lower	Upper				Lower	Upper				Lower	Upper	
Gender (male)	0.138	1.090	0.693	1.714	0.710	0.098	0.962	0.757	1.224	0.754	0.008	0.993	0.848	1.163	0.930
Race (white)	2.940				0.230	12.825				0.002	9.839				0.007
black	1.176	1.586	0.689	3.651	0.278	6.732	1.494	1.103	2.023	0.009	5.540	1.274	1.041	1.558	0.019
Other	0.376	0.597	0.115	3.099	0.540	12.509	1.968	1.352	2.864	< 0.001	9.374	1.525	1.164	1.998	0.002
Stage (IIIC)	3.563				0.313	5.014				0.171	5.744				0.125
IIB	1.448	0.331	0.055	2.005	0.229	2.925	0.469	0.197	1.117	0.087	2.614	0.648	0.383	1.096	0.106
IIIA	0.110	0.818	0.250	2.676	0.740	0.698	0.759	0.398	1.449	0.403	0.103	0.937	0.630	1.394	0.748
IIIB	0.037	0.898	0.301	2.683	0.848	0.068	0.947	0.630	1.425	0.794	0.090	0.960	0.736	1.253	0.764
T (T4b)	7.420				0.191	3.217				0.667	19.409				0.002
T1a	0.992	2.625	0.393	17.532	0.319	0.004	0.000	0.000	7.300	0.950	0.015	0.000	0.000	2.470	0.904
T1b	0.003	0.000	0.000	15.070	0.957	2.162	0.305	0.063	1.485	0.141	6.988	0.231	0.078	0.684	0.008
T2	0.009	1.074	0.236	4.899	0.926	1.023	0.630	0.258	1.541	0.312	9.566	0.426	0.248	0.732	0.002
T3	1.123	0.579	0.211	1.591	0.289	1.796	0.676	0.381	1.198	0.180	17.756	0.480	0.341	0.676	< 0.001
T4a	0.086	0.852	0.293	2.480	0.769	0.424	0.868	0.567	1.328	0.515	8.092	0.681	0.523	0.887	0.004
N (N3)	2.345				0.504	4.165				0.244	14.814				0.002
N0	0.090	1.322	0.214	8.153	0.764	0.466	0.746	0.322	1.729	0.495	9.652	0.462	0.284	0.752	0.002
N1	0.258	0.740	0.231	2.371	0.612	0.516	0.804	0.443	1.458	0.472	14.215	0.492	0.340	0.712	< 0.001
N2	0.314	0.733	0.247	2.171	0.575	3.657	0.689	0.470	1.009	0.056	9.112	0.675	0.523	0.871	0.003
Age (≤ 65)	5.084	0.669	0.472	0.949	0.024	0.875	0.894	0.708	1.130	0.350	52.995	0.552	0.470	0.648	< 0.001
Grade (I–II)	1.552	0.807	0.576	1.131	0.213	2.196	0.839	0.665	1.058	0.138	0.893	0.924	0.786	1.088	0.345

## DISCUSSION

In recent years, radiation therapy (RT) for resectable gastric cancer patients without distant metastases has remained disputed [13]. In our study, we evaluated which locoregional subsites benefited most from adjuvant RT combination therapy among gastric cancer patients without distant metastases, who had undergone complete resection of gastric cancer. We found that adjuvant RT significantly prolonged survival time in completely resected gastric cancer without distant metastases patients, especially patients with LN metastasis (N1, N2, N3), *primary tumor invasion (T2,T3*, T4a,T4b) *or locally advanced* (IIIA,IIIB,IIIC).The multivariate Cox proportional hazard regression analysis showed that radiation was an independent prognostic factor (“RPS”, hazard ratio (HR) = 0.797, 95% confidence interval (CI) 0.650–0.977, *p <* 0.05; “RAS”, HR = 0.515, 95% CI 0.448–0.592, *p <* 0.001).

In a review based on 9 studies, Valentini et al. reported that the 5-year survival of resectable gastric cancer patients statistically significant benefit with the addition of RT [14]. Recently, SUNG KIM et al. reported radiation treatment after D2-resected gastric-cancerpatients can decrease recurrence and prolong survival especially patients with IIIA and IIIB patients [15]. A meta-analyses also verified that Preoperative (chemo)radiotherapy might be associated with the significant improvement in overall survival compared with over surgery alone [16]. All these studies demonstrated that radiation treatment was a potential approach to improve the outcome of selected resectable gastric cancer patients without distant metastases. Recently, the opposite result was reported there was neither survival benefit no net medical cost advantage by adding RT in Gastric Cancer adjuvant treatment [17].

However, not all resectable patients without metastatic gastric cancer obtained a survival benefit from radiation.

Among those with stageT2, T4a,T4b,IIB,N0 and N1 cancer, no statistically significant differences were found in prolong survival time between RPS and no radiation groups (*p* > 0.05).

Cheng, J. *et al.* [18] reported that perioperative chemotherapy provided a significant improvement in OS compared to adjuvant chemoradiotherapy.

In N0 subgroup no radiation survival time is better than RPS, and no statistically significant differences were found in RAS.

INT 0116 study reported that adjuvant chemoradiotherapy after curative surgery in node negative patients provided a no significant improvement in OS (HR = 0.77, 95% CI: 0.46 to 1.30, *p* = 0.333) [7]. In a meta-analysis based on 13 studies reported that OS data for node positive patients were significant benefit with the use of RT (HR = 0.73, 95% CI: 0.62 to 0.86, *p* = 0.001) [19].

Multivariate analysis of overall survival by Cox proportional hazards modelling, we found that the survival improvement of patients in the radiation treatment groups was significantly higher compared with that of patients in the surgery only. It reported that the 5-year survival rates were consistently longer in the postoperative chemoradiotherapy group at Stages II, IIIA, IIIB, and IV than those in the surgery only group [15].

Some limitations may have influenced the results of this study. First, our study are inherent to the methodology of retrospective analyses, including selection bias and potential confounders. Because of insufficient sample capacity we integrated the data of the following patient groups to reduce bias: Grade I and grade II were integrated into the grade I–II subgroup, Grade III and grade IV were integrated into the grade III–IV subgroup. Second, information such as the chemotherapy status, which kind surgery was performed D1 or D2 gastrectomy, locoregional recurrence, radiation techniques, total doses, fractionations, radiation-related toxicity and comorbidities were not included in the SEER database.

Finally, the determination of the T-stage, N-stage tumor stage of patients who underwent surgery depended on the postoperative pathologic results, while for those RPS patients tumor stage at diagnosis were unclear.

In conclusion, we sought to evaluate whether resectable gastric cancer patients without distant metastases would benefit from radiation. The results showed that radiation treatment was able to improve effective survival time in T3, T4a, T4b, N1, N2, N3, IIIA, IIIB and IIIC cancer. RPS is different from RAS in special subgroup.

From the results of this study, we considered that patients who were younger and those with locally advanced stage primary tumors might obtain a greater survival benefit from radiation treatment than others.

Additionally, radiation therapy may strengthen the survival benefit that is gained from surgical treatment. Our study was a retrospective analysis with limitations and further prospective randomized trial are needed to validate our hypothesis.

## MATERIALS AND METHODS

### Patient selection

The Surveillance, Epidemiology, and End Results Program (SEER) database is an authoritative source of information on cancer incidence and survival, sponsored by the National Cancer Institute. In the SEER database, currently collects and publishes cancer incidence and survival data from 18 population-based cancer registries, which covers approximately 28 percent of the population in the United States.

The database of the SEER program includes information on patient demographics, race, sex, tumor histology, primary tumor site, stage at initial diagnosis, surgery, radiotherapy, and survival.

Our retrospective study contained 5744 patients from the SEER database (SEER*Stat 8.3.4) who were initially diagnosed with histologically confirmed gastric cancer without distant metastases, between 2010 and 2013. The characteristics of 5744 patients with gastric cancer without distant metastases in Supplementary Table 1. Histological type were limited to adenocarcinoma, mucinous adenocarcinoma, signet ring cell carcinoma. Inclusion criteria included only patients who underwent D2 surgical treatment with age of diagnosis more than 18 years; one primary only; cause of death dead due to cancer; surgery performed; without distant metastases; AJCC's TNM stage of the 7th edition. Exclusion criteria included patients less than 18 years of age; distant metastases and occult gastric cancer (no evidence of primary tumor). The IA, IB, IIA subgroups were eliminated considering the far smaller number of patients who underwent radiation. The remaining 3309 patients were divided into patients who underwent radiation after surgery (“RAS” group) and patients who underwent radiation prior to surgery (“RPS” group).

### Statistical analysis

Count data were analyzed by chi-square test. The median survival time (MST) and survival curves, were estimated with the Kaplan-Meier method, and the log-rank test was performed to evaluate survival in the different groups. Hazard ratios (HRs) along with 95% confidence intervals (CI) were calculated using the multivariate Cox proportional hazard regression model to determine the influences of other factors including age, race, gender, grade, histological type, T-stage, and N-stage, on survival. Statistical tests were two-sided, *p <* 0.05 was considered statistically significant. SPSS22.0 (SPSS Chicago, IL, USA) software was used for the statistical analysis.

## SUPPLEMENTARY MATERIALS TABLE


