# Supplementary material for: Outcomes of radiation therapy for resectable M0 gastric cancer
Source: Oncotarget. 2017 Nov 3;9(2):1726–34. doi: 10.18632/oncotarget.22574 (PMC5788594; doi:10.18632/oncotarget.22574)
Supplement: Supplementary file 1 [file oncotarget-09-1726-s001.pdf]

# Outcomes of radiation therapy for resectable M0 gastric cancer

## SUPPLEMENTARY MATERIALS

**Supplementary Table 1: The characteristics of 5744 patients with gastric cancer without distant metastases**

| Characteristic | Overall no. (%) (*N* = 5744) | Radiation prior to surgery | Radiation after surgery | No radiation | *p* value |
|---|---|---|---|---|---|
| **Age at diagnosis (y)** | 5744 | 731 (12.7) | 1348 (23.5) | 3665 (63.8) | < 0.05 |
| ≤ 65 | 2766 (48.2) | 484 (17.5) | 790 (28.6) | 1492 (53.9) | |
| < 65 | 2978 (51.8) | 247 (8.3) | 558 (18.7) | 2173 (73.0) | |
| **Gender, *N* (%)** | 5744 | | | | < 0.05 |
| Male | 3604 (62.7) | 596 (16.5) | 824 (22.9) | 2184 (60.6) | |
| Female | 2140 (37.3) | 135 (6.3) | 524 (24.5) | 1481 (69.2) | |
| **Race, *N* (%)** | 5699 | | | | < 0.05 |
| White | 3795 (66.1) | 650 (17.1) | 786 (21.7) | 2359 (62.2) | |
| Black | 684 (11.9) | 25 (3.7) | 217 (31.7) | 442 (64.6) | |
| Other | 1220 (21.2) | 54 (4.4) | 338 (27.7) | 828 (67.9) | |
| T stage * | 5606 | | | | < 0.05 |
| T1a | 757 (13.2) | 15 (2.0) | 25 (3.3) | 717 (94.7) | |
| T1b | 755 (13.1) | 23 (3.0) | 89 (11.8) | 643 (85.2) | |
| T2 | 736 (12.8) | 107 (14.5) | 171 (23.2) | 458 (62.2) | |
| T3 | 2184 (38) | 513 (23.5) | 621 (28.4) | 1050 (48.1) | |
| T4a | 887 (15.4) | 22 (2.5) | 342 (38.6) | 523 (59.0) | |
| T4b | 287 (5.0) | 19 (6.6) | 91 (31.7) | 17 (61.7) | |
| **Tumor grade** | 5364 | | | | < 0.05 |
| Well differentiated; Grade I | 350 (6.1) | 72 (20.6) | 58 (16.6) | 220 (62.9) | |
| Moderately differentiated; Grade II | 1535 (26.7) | 205 (13.4) | 352 (22.9) | 978 (63.7) | |
| Poorly differentiated; Grade III | 3350 (58.3) | 394 (11.8) | 820 (24.5) | 2136 (63.8) | |
| Undifferentiated; anaplastic; Grade IV | 129 (2.2) | 12 (9.3) | 32 (24.8) | 85 (65.9) | |
| **N-stage*, *N* (%)** | 5744 | | | | < 0.05 |
| N0 | 2605 (45.4) | 241 (9.3) | 286 (11.0) | 2078 (79.8) | |
| N1 | 1249 (21.7) | 323 (25.9) | 333 (26.7) | 593 (47.5) | |
| N2 | 865 (15.1) | 127 (14.7) | 310 (35.8) | 428 (49.5) | |
| N3 | 1025 (17.8) | 40 (3.9) | 419 (40.9) | 566 (55.2) | |
| AJCC tumor stage* | 5744 | | | | < 0.05 |
| IA | 1299 (22.6) | 35 (2.7) | 26 (2.0) | 1238 (95.3) | |
| IB | 545 (9.5) | 46 (8.4) | 99 (18.2) | 400 (73.4) | |
| IIA | 591 (10.3) | 27 (4.6) | 170 (28.8) | 394 (66.7) | |
| IIB | 904 (15.7) | 203 (22.5) | 253 (28.0) | 448 (49.6) | |
| IIIA | 860 (15.0) | 255 (29.7) | 238 (27.7) | 367 (42.7) | |
| IIIB | 793 (13.8) | 102 (12.9) | 300 (37.8) | 391 (49.3) | |
| IIIC | 752 (13.1) | 63 (8.4) | 262 (34.8) | 427 (56.8) | |

* T-stage , N-stage and AJCC tumor stage according to the 7th edition of AJCC TNM staging.
